# In a visual inverted pendulum balancing task avoiding impending falls gets harder as we age

**DOI:** 10.1007/s00221-025-06997-x

**Published:** 2025-01-16

**Authors:** Hannah E. Park, Avijit Bakshi, James R. Lackner, Paul DiZio

**Affiliations:** Ashton Graybiel Spatial Orientation Laboratory, Brandeis University, MS 033, 415 South Street, Waltham, MA 02453 USA

**Keywords:** Falling, Balancing, Serial Decision, Age-related Differences, Risk of Falling, Visual Inverted Pendulum (VIP)

## Abstract

Younger adults (YA) and older adults (OA) used a joystick to stabilize an unstable visual inverted pendulum (VIP) with a fundamental frequency (.27 Hz) of half that of bipedal human sway. Their task was to keep the VIP upright and to avoid ± 60° “fall” boundaries. Both age groups were tested with joystick gains and delays simulating age-related muscle strength and reflex slowing, respectively. In previous VIP and analogous self-balancing tasks, we observed a mixture of discrete corrective commands toward the balance point and destabilizing commands toward an impending fall. We hypothesized that (1) OA would fall more than YA, (2) traditional whole-trial stability and variability measures would differ across age groups and VIP conditions, and (3) different dynamics of corrective and destabilizing commands would discriminate falling from recovery. Results: (i) Traditional whole-trial performance metrics of fall incidence and the variance of position and velocity were worse in OA than YA and worse with longer delays and excessive joystick gains; (ii) OA made fewer corrective and more destabilizing commands than YA only when falling was imminent; (iii) when falls were imminent, a logistic model fit the percentage of inactive, corrective, and destabilizing commands as a function of time left to fall; and (iv) OA were like YA in switching between inaction and action, but exhibited less frequent and less prompt corrective commands than destabilizing commands relative to YA. We discuss whether such a decision-like process may also operate in a bipedal stance.

## Introduction

The control maneuvers evoked during continuous balancing of various unstable pendular systems have received considerable attention (Balasubramaniam 2013; Gawthrop et al. 2013; Vimal et al. 2016; Yoshikawa et al. 2016); however, the nature of control commands when the threat of a fall is imminent has not been distinguished well. We define three different regimes of balancing—no imminent threat of falling (*Safe*), escaping the imminent threat of falling (*Saved*), and succumbing to the imminent threat of falling (*Failed*). We employed a visual inverted pendulum (VIP) paradigm to discriminate non-reflexive corrective versus destabilizing movements, and we found that they have different incidence profiles across the three regimes, with magnified differences across age groups when falls were imminent.

Skillful manual tracking or nulling of an object’s motion, as well as bipedal nulling of postural sway, involve continuous proportional processes and discrete perceptual-motor processes (Craik 1947; Vince 1948; Morasso and Schieppati 1999; Loram and Lakie 2002; Lakie and Loram 2006; Loram et al. 2011; Gawthrop et al. 2014; Morasso et al. 2019). The former tends to work on small spatial and short temporal scales subserved by peripheral musculoskeletal, reflexive, and coordinative substrates (Bizzi et al. 1991; Kuo and Zajac 1993; Winter et al. 1996; Kurtzer et al. 2008; Bakshi et al. 2019), while the latter operate on relatively coarser clock-like or event-related spatiotemporal sample spaces (Bottaro et al. 2008; Bye and Neilson 2010) associated with more cerebral substrates (Vaillancourt et al. 2007; Grillner and Robertson 2016). For example, overlapping mixtures of continuous and discrete processes have been observed when humans stand bipedally (Bottaro et al. 2005), use ankle torques to balance a physical inverted pendulum of the same mass and center of mass height as a human body (Loram and Lakie 2002), or manually balance a physical or visually simulated pole (Cabrera and Milton 2004; Cluff et al. 2011; Zgonnikov and Markkula 2019).

Studying discrete, high-level processes is facilitated by segregating them from the continuous reflexive ones. Some progress has been made by studying how individuals seated in a motorized inverted pendulum simulator (multi-axis rotating system, MARS) stabilize their attitude by manipulating a joystick (Panic et al. 2015; Vimal et al. 2023). In these studies, the MARS was programmed to behave like an inverted pendulum toppling leftward or rightward about the approximate center of mass of a blindfolded individual seated within it, who is instructed to stay as close as possible to the vertical direction of balance (DOB) and to avoid ± 60° “fall” boundaries. The MARS joystick moves with the subject chair, producing no deflection or force feedback on the hand; consequently, no manual righting reflexes are elicited. The pertinent results are: first, blindfolded individuals undergoing roll toppling are able to balance themselves about the DOB; second, participants spontaneously adopt pulsatile rather than continuous proportional joystick commands; third, 10–20% of joystick commands are destabilizing, accelerating the MARS away from the DOB, rather than corrective, accelerating it towards the DOB. Parallel results are found when a joystick is used to balance a visual inverted pendulum (VIP) displayed on a computer monitor (DiZio and Davies 2022), where joystick commands are about 20% destabilizing. These results indicate that self-balancing and visual object balancing involve non-reflexive, discrete, destabilizing (anti-corrective) commands, which have not previously been reported or studied. Analogous destabilizing muscle responses during bipedal stance could be an under-appreciated mechanism of balance failure, including falling. We conducted an experiment to understand the trade-off of non-reflexive corrective versus destabilizing movements in the VIP task, using a methodology that could ultimately be applied to a natural bipedal stance.

**Fig. 1:**
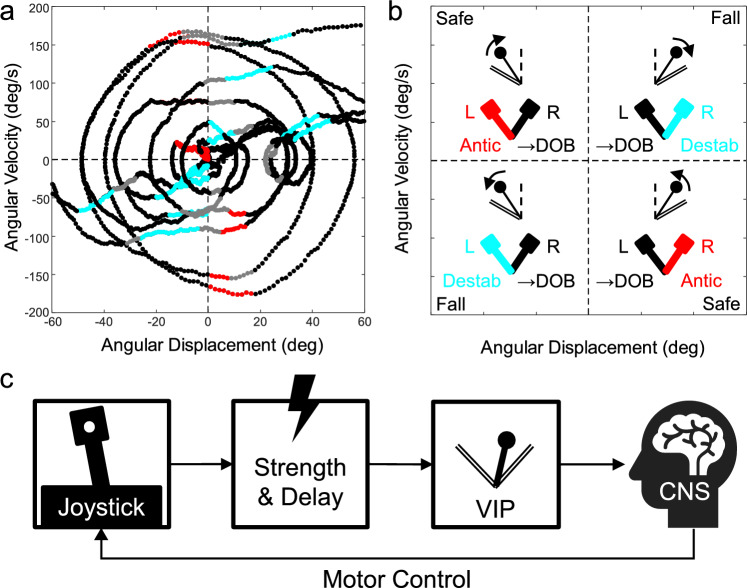
**a** Plot of pendulum angular velocity versus angular displacement from the direction of balance (DOB), of a typical younger adult’s VIP trial (30 s) with fall boundaries at ± 60°; there are 4 fall events to the right and 2 to the left. Samples are plotted in solid gray when the joystick is inactive, solid black when joystick commands accelerate the pendulum towards the DOB (corrective), cyan dots when commands accelerate the pendulum away from the DOB and towards a fall boundary (destabilizing), and red dots for commands which decelerate the pendulum as it approaches the DOB (anticipatory). **b** The same phase plane is divided into quadrants, each containing pictographs of the pendulum (top) and joystick (bottom). Quadrants Q2 and Q4 are labeled Safe because the pendulum is moving (arrows) toward the DOB (dashed line), and quadrants Q1 and Q3 are labeled Fall because the pendulum is moving toward the fall boundary (double line). Joystick deflection directions are color-coded the same as in panel a: corrective (black) accelerating the pendulum toward the DOB, anticipatory (red) decelerating the pendulum approaching the DOB, and destabilizing (cyan) accelerating toward a fall boundary. L = left and R = right. **c** The VIP control diagram

The experimental hypotheses and design aimed to understand the dynamics of the VIP balancing illustrated in Fig. 1. Figure 1a plots sampled VIP velocity versus position, with dot colors encoding functional types of joystick commands, which are illustrated in Fig. 1b, where the upper right (Q1) and lower left (Q3) quadrants are designated “Fall,” and the upper left (Q2) and lower right (Q4) quadrants of the same coordinate system are labeled “Safe.” In the Fall quadrants, the pendulum is leaning and moving toward a programmed boundary analogous to the ground plane in a bipedal stance; in the complementary Safe quadrants, the pendulum is moving toward the upright DOB. Entering a Fall quadrant can end in either a fall or an escape to the Safe quadrant; entering a Safe quadrant can end in transitions to Fall quadrants but not in a fall. In other words, falls are less imminent in the Safe quadrants. In the Fall quadrants of Fig. 1a, the dots are gray when the joystick is inactive (deflected less than 1°), black when active deflection accelerates the pendulum towards the DOB (corrective), and cyan when the deflections accelerate the pendulum towards a fall boundary that it is already approaching (destabilizing). In the Safe quadrants, gray and black represent joystick inactivity or acceleration towards the DOB, respectively, and red indicates joystick commands producing acceleration away from the DOB. Red dots might be interpreted as anticipatory—keeping the pendulum from overshooting the DOB—and the black dots represent further accelerating the pendulum approaching towards the DOB. The distinction between these two types of commands (black and red) in the Safe quadrants has been observed previously, but the distinction between corrective and destabilizing movements (black and cyan) in the Fall quadrants has not been conceptually or empirically distinguished in previous literature.

Our experiment was constructed to compare performance and commands between the Safe and the Fall quadrants versus traditional whole-trial analysis. We compared the performance of young adults (YA) and older adults (OA) (Liao et al. 1997) under VIP conditions which simulate documented effects of aging on the loss of muscle strength and increased reflex latency in related tasks (Nadler et al. 2002; Simoneau et al. 2007; Sosnoff and Newell 2007; Narici et al. 2008; Hasson et al. 2011). We expected OA to fall more and to show decrements in traditional whole-trial stability and variability measures, similar to what has been reported in a range of other eye-hand tasks (van Wieringen et al. 2022), as well as in stance and gait (Tinetti et al. 1986; Howcroft et al. 2017). We expected the dynamics of discrete joystick commands in the Fall quadrants to have a larger impact than in the Safe quadrants on performance under challenging conditions, because the time to contact a fall boundary is shorter in the Fall than in the Safe quadrants. For the same reason, we expected to find age differences in the Fall quadrants.

## Materials and methods

### Participants

Participants were recruited from Brandeis University and the Greater Boston area. Individuals were excluded if they reported muscular or skeletal impairments, neurological conditions affecting movement or mental state, cognitive impairments, and sensory conditions affecting balance, hearing, touch, or vision. In addition, OA were also excluded if they reported inability to stand and walk independently for 60 s without falling. Fifty-seven OA and YA participated, composed of: (1) 30 YA, aged 18–29 (22.53 ± 3.59 *SD*, 18 females, 5 left-handed); and (2) 27 OA, aged 60–78 (66.04 ± 4.85 *SD*, 14 females, 1 left-handed). One OA participant was excluded as an outlier after data collection, so 26 OA were analyzed. All participants gave written consent to the experimental protocol approved by the Brandeis University Committee for the Protection of Human Subjects; all were compensated for their time.

### Apparatus

Figure 1c is a diagram of the test setup. An image of a mass on top of a stick that could rotate about its base was rendered at 50 Hz on a 210 × 210 dpi, 75 Hz scan rate monitor placed at a comfortable distance about two feet from the participant’s midline. Fall boundaries set to ± 60° from the vertical direction of balance (0°, DOB) were always displayed on the screen. Exceeding the fall boundary paused a trial. To drive the VIP, participants used their dominant hand to deflect a joystick (CH Flightstick Pro USB 10-bit) fixed on the tabletop, which could tilt ± 30° laterally with a slight elastic attraction to its central null position. The joystick and pendulum states were sampled (and stored) at 200 Hz, and fed to a MATLAB function (ODE45), solving the differential equation for the driven pendulum: $\ddot{\uptheta }= {K}_{P}\text{sin}\theta +{K}_{J}\varphi$, where θ is the pendulum angular displacement from the vertical, $\ddot{\uptheta }$ is angular acceleration, K_P_ governs the intrinsic pendulum toppling rate, φ is the joystick angle, and K_J_ quantifies the gain of the joystick drive. The value of K_P_ was always 171.9°/s^2^, equivalent to 0.27 Hz, while the fundamental frequency of human sway is about 0.5 Hz. This K_P_ value had been found in pilot studies to be challenging but not frustrating for both age groups. Every participant experienced two joystick gain conditions: K_J_ = 9.5 and 19.1 s^−2^. These values were chosen based on extensive (unpublished) parametric testing of the VIP paradigm, which had shown that a constant relationship between K_J_ and K_P_ provides optimal task level performance – fewest falls and lowest position/velocity variance; setting K_J_ too low made full joystick deflection too weak to save the pendulum approaching a fall boundary, and K_J_ too high resulted in operator-induced oscillations. For our chosen value of K_P_ = 171.9°/s^2^, the optimal K_J_ is 9.5 s^−2^. Under the premise that joystick gain simulates effective muscle strength, we henceforth designate K_J_ = 9.5 s^−2^ as “normal” and K_J_ = 19.1 s^−2^ as the “hyper” gain/strength condition. The equation of motion could compute the next pendulum state fast enough to achieve no lag between joystick deflection and the rendered pendulum response. In some trials, we added 30 and 60 ms delays between reading the joystick and feeding it to the simulation algorithm, to effectively increase the end-to-end sensorimotor delays. Sixty ms was chosen as the maximum experimental delay because it is the approximate maximum published estimate of age-related intrinsic visuomotor delay. We reasoned that delays in this range might make YA subjects perform progressively more like OA with only their intrinsic delay (0 experimental delay), and make OA performance deteriorate more rapidly, especially under hyper gain conditions.

### Procedure

Before starting the experiment, participants viewed an introductory video and completed 3 practice trials. They were told to use the joystick to keep the inverted pendulum at the DOB and avoid falls. A fall occurred when the pendulum reached one of the ± 60° boundaries; the pendulum then would disappear, and the trial would not resume until the joystick was returned to its central neutral position and the joystick’s trigger button was pressed. The pendulum was near the DOB and near 0 velocity at the beginning of trials and after resets. Eighteen trials were completed, each lasting 30 s, excluding fall reset time. Every participant completed 3 repetitions of 6 randomized factorial combinations of the 3 joystick command delays (0, 0.03, and 0.06 s) and 2 joystick gains (9.5 and 19.1 s^−2^). Rests were given based on participants’ requests.

### Data reduction

*Whole-trial measures of task-level VIP performance*. Three dependent variables (DV) quantifying task-level performance were calculated for each trial: (i) number of falls (#Falls) defined as the number of times the VIP reached the ± 60° fall boundaries; (ii) pendulum angular position variability (sIQR_Pos_), defined as the semi-interquartile range of angular position, in degrees; and (iii) pendulum angular velocity variance (sIQR_Vel_), defined as the semi-interquartile range of angular velocity, in degrees per second. Each DV was averaged across the three repetitions of each factorial condition for each participant.

*Whole-trial measures of command types*. We identified every time sample as belonging to one of the four types of joystick control maneuvers mentioned in Fig. 1—(i) inactivity (**I**): when the joystick was in the neutral inactive position, defined as a ± 1° band around 0° of joystick deflection^1^; (ii) corrective reactions (**CR**): when joystick deflections opposed the falling motion of the pendulum, including decelerating it when approaching the nearest fall boundary (Fall quadrants) and accelerating it when approaching the upright (Safe quadrants), defined as when the sign of joystick displacement was opposite that of pendulum displacement; (iii) anticipations (**A**, Safe quadrants only): when joystick deflections decelerated the pendulum as it approached the upright, defined as when the sign of pendulum velocity was opposite that of both pendulum and joystick displacement; and (iv) destabilization (**D**, Fall quadrants only): when the pendulum was approaching the nearest fall boundary and joystick deflections accelerated it, defined as when pendulum displacement, pendulum velocity, and joystick displacement had the same sign and exceeded ± 1°. We then calculated the percentage of samples in a whole 30 s trial that fit the definitions of joystick inactivity (%Tot_I_), corrective reactions (%Tot_CR_), anticipations (%Tot_A_), or destabilizing commands (%Tot_D_), averaged over repetitions per participant.

*Measures of the dynamics of command maneuvers as a function of pendulum position*. Every sequence of pendulum samples was given one of three balancing designations defined by how it exited the Safe or Fall quadrants illustrated in Fig. 1a and b—(i) *Safe* balancing: all VIP epochs spent in a Safe quadrant (Q2 or Q4) moving towards the upright DOB with no immediate risk of falling, because the only possible fate is transition to a Fall quadrant; (ii) *Saved* balancing: VIP sequences in a Fall quadrant (Q1 or Q3) moving towards a fall boundary and eventually escaping to a Safe quadrant; and (iii) *Failed* balancing: VIP sequences in a Fall quadrant (Q1 or Q3) moving towards and culminating in a fall. For each of these three balancing regimes, we computed the percentage of each type of command maneuver present as a function of the angular displacement of the pendulum from the DOB, collated for all trial repetitions and conditions together. Note that D is only defined in *Saved* and *Failed* balancing (quadrants Q1 and Q3), A is only defined in *Safe* balancing (quadrants Q2 and Q4), while CR and I are defined in all three balancing regimes. Preliminary visual inspection of Fig. 3 showed monotonic increases of CR, decreases of I, and decreases of A and D with respect to the pendulum displacement. Each monotonic command type trace was quantified by three DVs: (i) area under the percentage curve,${AUC}_{P}^{I/CR/A/D}$,^2^ (ii) percentage at the DOB, ${\%@DOB}_{P}^{I/CR/A/D}$, and (iii) the pendulum position where the command percentage first reached its extreme difference from the percentage at the DOB (maximum for CR, minimum for I, A, and D), ${\theta ext}_{P}^{I/CR/A/D}$.

**Fig. 2:**
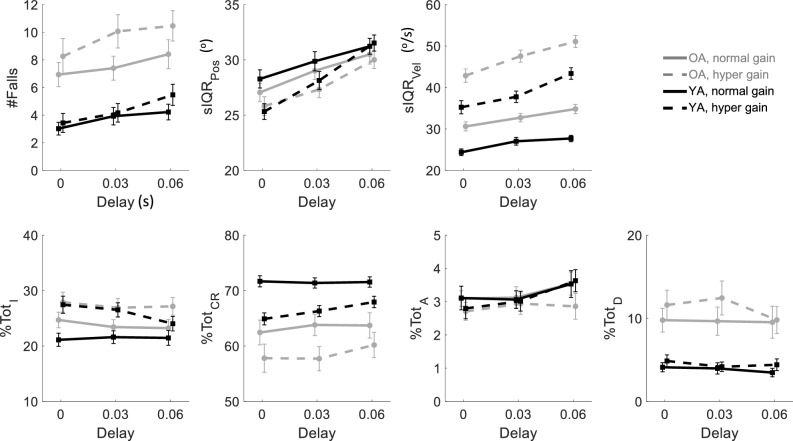
The average magnitude of dependent variables computed for the whole trial as a function of VIP conditions (joystick gain and delay). In each plot, the abscissa represents the delay, while the gains are represented by different lines (solid for normal and dashed for hyper), OA are in gray and YA in black. Error bars represent ± 1 standard error. The top row presents VIP performance measures and the lower row presents the percentage of the four types of joystick commands defined in the text: I = inactivity, CR = corrective reactions, A = anticipatory, and D = destabilizing

### Statistical analysis

All statistics were done using the MANOVA.RM package in RStudio (Friedrich et al. 2019) to conduct resampling-based hypothesis testing. Our focus was on determining the effect of age and the interactions of various factors with age. We first conducted a multivariate analysis of variance (MANOVA) on all DVs using the modified ANOVA-type statistic with 10,000 wild bootstrap routine runs. The MANOVA statistics are not reported below, but follow-up univariate ANOVAs with the same resampling were only conducted on independent variables for which the MANOVAs revealed significant main or interaction effects. The criteria for univariate ANOVA significance were either the ANOVA-type statistic (ATS) for mixed design analyses or the Wald-type statistic (WTS) for one-way analyses. These parametric analyses are robust to violations of the assumptions for standard MANOVAs and ANOVAs. Bonferroni corrections were made to prevent inflating Type 1 errors across multiple DVs. Any ANOVA or pairwise comparison result not reported may be considered non-significant.

## Results

### Whole-trial measures of task-level VIP performance

The top row of plots in Fig. 2 displays the performance variables by age group and VIP conditions. A 3-way mixed MANOVA (age, VIP gain, and VIP delay) found significant age by gain interactions and main effects of age, gain, and delay for #Falls, sIQR_Pos_, and sIQR_Vel_. Subsequent individual 3-way mixed ANOVAs on each performance measure, with Bonferroni correction criterion of *p* = 0.017, showed no significant interaction of age with gain or delay. However, there was a significant age main effect for #Falls (ATS = 13.70, *p* < 0.001), main effects of delay for all DVs (#Falls: ATS = 33.85; sIQR_Pos_: ATS = 38.75; sIQR_Vel_: ATS = 50.31; all *p* < 0.001), and of gain for all DVs (#Falls: ATS = 16.56, *p* < 0.001; sIQR_Pos_: ATS = 7.99, *p* = 0.006; sIQR_Vel_: ATS = 167.57, *p* < 0.001). Finally, comparisons between short and long delays to the control level delay were significant in all variables (*p* < 0.001; Wilcoxon signed-rank tests with Bonferroni correction, *p* = 0.025). Thus, from a whole-trial level of analysis, we found that each measure showed distinct performance changes with increased age, gain, and delay. #Falls were increased by age, artificially high gain, and larger delay; both sIQR_Pos_ and sIQR_Vel_ showed no age effect but increased with hyper gain and longer delay.

### Whole-trial measures of command types

Our next goal was to determine whether the percentages of each command type over entire trials were affected by age and VIP conditions, Fig. 2, bottom row. A 3-way mixed MANOVA (age, gain, and delay) on the four command types showed the main effects and interactions for all factors. Subsequent univariate 3-way mixed ANOVAs with Bonferroni correction criteria of *p* = 0.0125 showed that an age main effect was significant for CR and D (%Tot_CR_: ATS = 12.30, *p* < 0.001; %Tot_D_: ATS = 13.87, *p* < 0.001). The main effect of gain was significant for I, CR, and D (%Tot_I_: ATS = 72.55, *p* < 0.001; %Tot_CR_: ATS = 141.76, *p* < 0.001; %Tot_D_: ATS = 9.37, *p* = 0.003). The main effect of the delay was only significant for A (%Tot_A_: ATS = 7.79, *p* = 0.001). Thus, from a whole-trial level of analysis, increased age and gain increased performance-degrading D commands and decreased performance-enhancing CR command types, while I commands were only affected (increased) by the gain increase. Age by VIP condition interactions were not significant in any univariate analysis.

### Dynamics of command maneuvers as a function of pendulum position

**Fig. 3:**
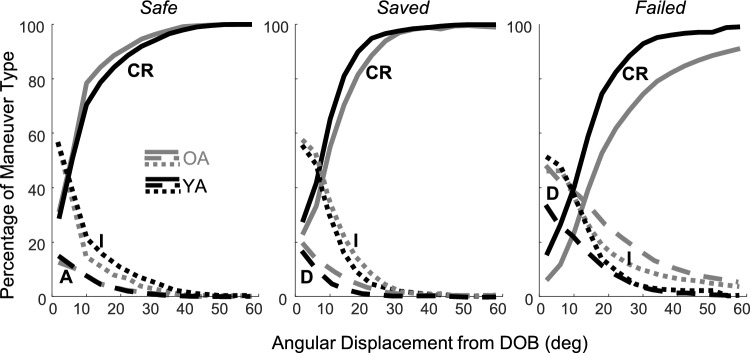
Joystick maneuvers as a function of the pendulum angular position, separately in three balancing regimes defined in the text: *Safe*, *Saved*, and *Failed*. OA are shown in gray and YA in black, and labels and line styles distinguish command types (I = dotted, CR = solid, A or D = dashed). For *Safe* and *Saved* balancing, the evolutions of all command types across pendulum positions are the same across age groups. However, in the *Failed* regime, near irrecoverable falls, major age-dependent degradation is apparent for OA—they make fewer corrective and more destabilizing commands

Because of the lack of age interactions with gain and delay for whole trial univariate analyses of command percentages, the VIP gain and delay conditions were combined for our next objective—to compare the command maneuvers in the three balancing regimes. Figure 3 shows the percentage of command maneuvers as a function of pendulum position^3^ in *Safe, Saved*, and *Failed* balancing, by age group. For all three regimes, the position can span anywhere between DOB at 0° and the boundary at 60°; however, the velocity is always directed towards DOB in the *Safe*, and towards the boundary in *Saved* and *Failed* regimes. Only the I and CR commands are included in this analysis because they are the only command types that exist across all three balancing regimes. In Fig. 3, the greatest age differences are visible in the *Failed* regime and the least in the *Safe* regime. A 2-way mixed MANOVA on the three CR DV’s (${AUC}_{P}^{CR}$, ${\%@DOB}_{P}^{CR}$, and ${\theta ext}_{P}^{CR}$) showed main effects of age and regime, and their interaction. Subsequent univariate 2-way mixed ANOVAs, after Bonferroni correction, showed significant (*p* = 0.002 at least) main effects of age on ${AUC}_{P}^{CR}$ (ATS = 15.73) and ${\theta ext}_{P}^{CR}$ (ATS = 9.68). Regime main effects were significant (all *p* < 0.001) in all three measures of CR: ${AUC}_{P}^{CR}$ (ATS = 66.02), ${\%@DOB}_{P}^{CR}$ (ATS = 65.85), and ${\theta ext}_{P}^{CR}$ (ATS = 28.32). The age by regime interaction effect was significant on ${\theta ext}_{P}^{CR}$(ATS = 14.48, *p* = 0.006). The interaction effect was unpacked in two ways with pairwise comparisons. All three CR variables were significantly greater in YA than OA in *Failed* balancing (*p* ≤ 0.004 at least), but there were no age differences in *Safe* and *Saved* balancing. This means YA made a higher percentage of CR movements than OA at the beginning, middle, and end of only the *Failed* balancing regime. In addition, all three CR variables differed between the *Failed* and *Saved* regimes but not between *Saved* and *Safe*; CR was more prevalent and earlier in *Saved* than *Failed* (*p* < 0.001 for OA, *p* = 0.002 for YA). A comparable MANOVA analysis as above for the three I DVs showed a significant interaction effect between age and regime, with the main effect of regime but no main effect of age. Subsequent 2-way mixed ANOVAs on each I variable, after Bonferroni correction, showed no age main effects or interactions; however, the means of %I as a function of pendulum position showed a trend similar to the significant interaction found for %CR – OA were visibly more inactive than YA in the *Failed* but not in the *Safe* or *Saved* regimes.

Significant age differences in %CR specific to *Failed* balancing could either emerge from differences generated within that regime or could be due to differences in how participants entered the Fall quadrant from a Safe quadrant. Fall-to-Safe quadrant transitions happen when passing through exactly 0 velocity, but Safe-to-Fall quadrant transitions occur at a finite velocity. We found no age differences in the speeds of Safe-to-Fall quadrant transitions (*t* (54) = 1.91, *p* > 0.05): OA = 57.48°/s, YA = 50.85°/s. This together with Fig. 3, suggested that age differences were being generated *within* the Fall quadrants, particularly in the *Failed* regime.

### Dynamics of command maneuvers as a function of time left to fall (TLTF)

**Fig. 4:**
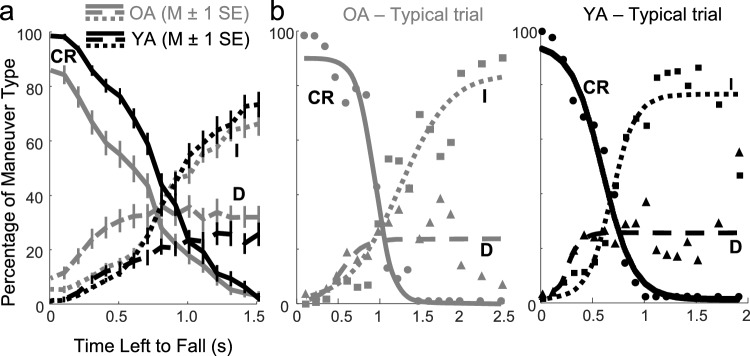
**a** Average across participants (± 1 standard error) of joystick maneuvers as a function of TLTF for the *Failed* regime. The age groups and the I, CR, and D traces are labeled and depicted as in Fig. 3. For I, there was little age-dependent difference. In general, OA made fewer CR commands relative to YA across all TLTF, and the differences were particularly amplified as the TLTF diminished, below about 0.8 s. OA also made more wrong choices (D) across all TLTF than YA. **b** Percentage of joystick maneuvers as a function of TLTF in the *Failed* balancing regime for single trials for typical OA and YA participants. Empirical values are shown with different markers for I, CR, and D (I = squares, CR = circles, and D = triangles), and their logistic curve fits are shown with different line styles

All analyses up to this point were planned a priori, but the finding that age differences in command structure were significant *only* in *Failed* balancing motivated further post-hoc age group comparisons of the command structure preceding falls. In *Failed* balancing, falling is imminent and TLTF is calculable at every sample, for I, CR, and D commands (A commands are not present in *Failed* balancing). We computed TLTF for every *Failed* balancing sample by simulating the pendulum trajectory starting at the current state until it reached the fall boundary, with the joystick input set to zero. We binned the TLTFs (0.01–2.385 s) into 0.1 s increments and plotted the percentage of I, CR, and D as a function of the TLTF bin. These curves, averaged across participants and VIP conditions, are plotted in Fig. 4a. Note that these curves are left–right reversed relative to Fig. 3 because TLTF = 0 (the left side of the x-axis in Fig. 4a) corresponds to being at the fall limit (the right side of the x-axis in Fig. 3). The profiles of I commands for both age groups appear to be identical in *Failed* balancing. However, age differences are visible in the CR and D maneuvers: CR appear to occur earlier and more frequently for YA than OA, while D was less prevalent for YA than OA.

To quantify the percent of each maneuver type as a function of TLTF in the *Failed* balancing regime, we fitted nine distinct functions, each with 2 to 4 parameters, to the I, CR, and D curves for each trial and participant. We used the Bayesian Information Criterion (BIC) to compare the fitness of these models. When comparing multiple competing models, BIC is recommended (Field et al. 2012) as a method for model selection that is more conservative in adding parameters. BIC penalizes model complexity and favors simpler models, striking a balance between goodness-of-fit (least residual errors) and simplicity (fewer parameters), avoiding overfitting. For each fitted function, per age and command type, in Table 1, the number outside the parenthesis shows the rank ordering obtained in terms of BIC value, and the BIC value and r^2^ are shown in parenthesis. For both age groups, BIC identified the logistic function as the best fit for %I and %CR maneuvers among all fitted functions. For %D, the logistic function ranked the best for YA and was second to the double exponential for OA. Altogether, because the logistic function was the best fit for five of the six combinations of control type and age group, and the second-best fit for the remaining combination, we chose it for fitting all curves.

**Table 1:** Summary of model fits to the per-trial profiles of the percentage of each command type as a function of time left to fall (TLTF) in the Failed regime. The nine models fit are labeled in the header row, with the number of model parameters in parentheses. For each age group and command type, the number before the parenthesis is the relative ranking of each model, where 1 = best and 9 = worst, based on the obtained Bayesian Information Criterion (BIC) value, which tends to favor simpler models (fewer parameters) in the trade-off with model fitness. The first ranks are bolded. Within parentheses, the averaged BIC and r^2^ value for each model (BIC, r^2^) are shown. The logistic function was the best fit among all nine functions for I and CR, and for D, it was the second-best for OA (italicized) and the best for YA

	Linear (2)	Exponential (2)	Logarithmic (2)	Sqrt (2)	Quadratic (3)	Logistic Lo to Hi (3)	Logistic Hi to Lo (3)	Double Exponential (3)	Double Exponential (4)
%I_OA_	3 (88.6, 0.67)	8 (103.0, 0.31)	6 (98.9, 0.45)	4 (88.9, 0.60)	2 (83.4, 0.80)	**1 (75.0, 0.82)**	9 (115.3, − 0.06)	7 (99.2, 0.54)	5 (97.2, 0.50)
%CR_OA_	6 (91.2, 0.79)	9 (126.2, − 0.51)	7 (96.9, 0.62)	4 (85.3, 0.74)	2 (76.1, 0.92)	8 (123.2, −0.05)	**1 (62.3, 0.95)**	5 (85.5, 0.84)	3 (82.0, 0.81)
%D_OA_	5 (81.0, 0.30)	8 (89.8, − 0.61)	6 (81.5, 0.26)	4 (80.2, 0.28)	3 (78.4, 0.46)	*2 (77.7, 0.42)*	9 (91.3, 0.01)	7 (83.8, 0.25)	**1 (76.1, 0.46)**
%I_YA_	3 (104.3, 0.64)	7 (113.7, 0.44)	8 (114.2, 0.46)	4 (105.0, 0.63)	2 (98.7, 0.76)	**1 (82.3, 0.79)**	9 (130.3, − 0.05)	6 (112.6, 0.55)	5 (108.9, 0.54)
%CR_YA_	4 (105.4, 0.80)	9 (148.4, − 1.14)	7 (118.6, 0.60)	3 (104.4, 0.80)	2 (94.0, 0.89)	8 (141.7, − 0.09)	**1 (71.2, 0.94)**	5 (112.7, 0.67)	6 (112.9, 0.58)
%D_YA_	5 (85.2, 0.38)	8 (88.9, 0.23)	7 (88.3, 0.33)	4 (85.2, 0.39)	3 (81.2, 0.55)	**1 (65.4, 0.50)**	2 (80.9, 0.01)	9 (89.4, 0.34)	6 (85.6, 0.49)

Figure 4b illustrates the fitted curves and empirical percentage of each command type for typical OA and YA trials in the *Failed* balancing regimes. To compare the age groups for each maneuver type as a function of TLTF, six DVs were derived from the logistic fits, and divided into two categories. The first category corresponded directly to the three logistic parameters: i-iii) the relative height between the start and end points (${H}_{TLTF}^{I/CR/D}$), the temporal mid-point (${Tmid}_{TLTF}^{I/CR/D}$), and the logistic growth rate or steepness (${S}_{TLTF}^{I/CR/D}$). The second category included three variables analogous to those computed for characterizing the empirical curves of command maneuver type percentage as a function of pendulum position: iv-vi) area under the curve (${AUC}_{TLTF}^{I/CR/D}$), the percentage at the minimum TLTF (${\%@min}_{TLTF}^{I/CR/D}$), and the percentage at the maximum TLTF (${\%@max}_{TLTF}^{I/CR/D}$). Significant age-dependent differences were found in MANOVAs both on the three logistic parametric DVs and the three analogous variables. Univariate ANOVAs showed significant age differences in ${Tmid}_{TLTF}^{CR}$ (WTS = 8.76, *p* = 0.005), ${AUC}_{TLTF}^{CR}$ (WTS = 13.94, *p* < 0.001), and in ${\%@min}_{TLTF}^{CR}$ (WTS = 14.85,* p* < 0.001). YA transitioned to a higher proportion of CR earlier (with more time left to fall) than OA. They not only had a higher percentage of CR commands across the entire *Failed* balancing regime than OA, but YA also ultimately magnified the proportion of CR commands compared to OA right before a fall. In addition, ${\%@min}_{TLTF}^{D}$ and ${\%@min}_{TLTF}^{I}$ were both significantly higher for the older group (WTS = 12.95, *p* < 0.001; WTS = 10.29, *p* = 0.001).

In summary, (1) the absence of any age differences between pendulum velocity and I, CR, and D commands at entry to Fall quadrants (maximum TLTF) means both age groups enter the Fall quadrant performing similarly; (2) the significantly smaller ${Tmid}_{TLTF}^{CR}$ found in OA than YA means that when about to fall, OA made a slower transition than YA to dominance of CR relative to I or D; (3) the significantly lower ${AUC}_{TLTF}^{CR}$ in OA than YA means that before their eventual fall CR was less prevalent in OA than YA over the entire range of TLTF; and (4) smaller ${\%@min}_{TLTF}^{CR}$ and larger ${\%@min}_{TLTF}^{I}$ and ${\%@min}_{TLTF}^{D}$ in OA than YA means OA executed fewer CR relative to I or D than YA only when a fall was most imminent.

## Discussion

This study was designed to understand higher-level processes in balancing skills, evident in the decision-like switching between a finite set of manual command alternatives for balancing oneself or an external object (Loram and Lakie 2002; Cabrera and Milton 2004; Bottaro et al. 2008; Panic et al. 2015; Zgonnikov and Markkula 2019; Vimal et al. 2020; Wang et al. 2022). Our approach included (1) the use of the VIP task, which depends little on musculoskeletal biomechanics, reflexive stiffness and coordination, and vestibular/proprioceptive acuity, but more heavily expresses higher-level factors in sensorimotor skill, (2) a novel conceptual framework that emphasizes the spatiotemporal regimes where command timing and accuracy are critical, and (3) comparisons across age groups where balancing performance is known to decline in a manner that depends on both peripheral factors (Nardone et al. 1995; Richardson et al. 2014; Jahn 2019) and central sensorimotor integration and decision-making (Muller et al. 2004; Li et al. 2018).

*Effects of age, joystick gain, and delay across whole trials*. Our finding that YA outperform OA on whole-trial measures of VIP balancing is consistent with the results of comparable tests (Jagacinski et al. 1995; Liao et al. 1997). YA fell (contacted the fall boundaries) less often and oscillated with lower rates. In addition*,* YA fell more softly (with a lower average velocity ≈ 77 ± 18°/s) than OA (≈ 108 ± 33°/s), *p* < 0.001, W = 617, Wilcoxon rank-sum test. Our significant main effects of both gain and delay are consistent with previous conclusions that reflex force and latency are risk factors in self-balancing and visuo-manual tracking (Jagacinski et al. 1995; Nardone et al. 1995; Liao et al. 1997; Richardson et al. 2014; Jahn 2019). As expected, our artificially long delays increased the fall rate and the variance of VIP sway magnitude and rate. We also expected performance to be degraded by joystick gain increases because extensive parametric testing of the VIP paradigm had shown that the lower of our two gain conditions was optimal while the hyper gain elicited operator-induced oscillations. The artificially high joystick gains increased the fall rate and the variance of the VIP sway rate, paralleling the expectation of muscle strength being a factor.

In addition, the full pattern of joystick gain and delay effects of our experimental manipulation of VIP extend past studies, as they also rule out a *purely* peripheral explanation of performance. The maximum delay we imposed – 60 ms – is longer than any published estimate of age-related intrinsic visuomotor delay, so a purely peripheral explanation would predict worse performance in YA with a 60 ms delay than in OA with 0 experimental delay plus less than 60 ms intrinsic delay, especially under hyper gain conditions. However, YA with 60 ms joystick delays fell less than OA with no delay and comparable joystick gain (Fig. 2, top left). Our whole-trial results also extend the literature by showing that D commands, which have not been reported in previous object balancing studies, are present for both age groups, with a prevalence of about 5% in YA and 10% in OA (averaged across whole trials). Furthermore, I, CR, and D commands are unaffected by joystick delay but are reciprocally affected by age: OA > YA for I and D, and OA < YA for CR. By contrast, A commands are unaffected by age but increase with delay. The fact that A commands are not affected by age implies OA and YA do not differ in capacity for predicting pendular behavior. This separability of factors affecting I, CR, and D vis-à-vis A in whole-trial analysis, combined with the fact that I, CR, and D are definable in Fall quadrants but A only in Safe quadrants, suggest that balancing dynamics—task-relevant co-evolution of pendulum (plant output) and joystick (controller) states—are factors in balancing performance.

*Age differences in control maneuver types depend on dynamic regimes of balancing*. The absence of interaction effects among age, joystick gain, and delay factors on command types in whole-trial univariate analyses led us to combine different gain and delay conditions for each balancing regime. Additionally, we reasoned that command switching would be governed by time pressure, which would increase when falls are imminent. For the task difficulty (K_P_) we employed, the whole-trial rate of switching between the four functional command types was roughly 2 Hz, manifested as the number of color-coded command sub-sequences in the typical 30 s trial plotted in Fig. 1a, without the transitions caused by the pendulum crossing a quadrant. We partitioned the time series of pendulum balancing into three distinct dynamic regimes: *Safe, Saved,* and* Failed*, which are defined by time pressure and command selection. In the *Safe* regime, the control could be either I, CR, or A; in the *Saved* and *Failed* regimes, control could involve I, CR, or D maneuvers.

The overall evolution of all command types for both age groups exhibited monotonic transitions as a function of the angular deviation of the pendulum from the DOB. Figure 3 illustrates that for balancing in all regimes, the percentage of CR commands transitioned smoothly from a minimum near the DOB to a maximum when displaced 60° (at the fall boundary^4^). The percentage of I and D commands evolved in reverse, from high to low. In other words, **consistent patterns of command switching are observed with increases with deviation from the upright goal, which has never been reported before, and it occurs for both age groups**. Furthermore, age-related differences in the evolution of CR commands exist only in *Failed* balancing. YA made significantly more and earlier CR than OA only in the *Failed* balancing regime. Near the boundary, YA also made significantly fewer D movements and were less inactive than OA only in the *Failed* regime*.* These age group differences in novel patterns of command switching within the *Failed* balancing regime parallel age differences in falling; the group with fewer CR and more D commands (only in the *Failed* balancing regime) falls more often (falls occur only in *Failed* balancing). **We found no age differences in the speeds at which the age groups enter the Fall quadrant, meaning that age differences evolve *within* the *Failed* balancing regime**.

*Age-dependent, regime-specific, and time-critical switching between balancing command alternatives*. The observation that the age differences in command switching as a function of pendulum position occurred only in the dynamic regime where falls happen, and were reminiscent of logistic functions, motivated post-hoc analyses probing whether switching is time-critical. Guided by psychophysical and neurophysiological switching patterns for saccadic decisions (Schall and Thompson 1999; Salinas et al. 2010; Costello et al. 2013), we assessed whether VIP command switching improves or declines as the time before a fall shortens, leading to less time available for decision-making. This would predict that, in *Failed* balancing, (1) the rate of inactivity (I) should decrease (because something must be done) as the pendulum gets closer in time to contacting a fall boundary, and after the threshold for action is exceeded, (2) YA, who perform better, should switch faster and more successfully (increase CR and decrease D) than OA. We computed command probability as a function of time left to fall (TLTF), assessed logistic curve fits, and compared the fits with functionally relevant summary statistics. Logistic functions were the best fits (ranked 1st among 9 plausible monotonic functions) for I and CR for both age groups, and for D, it was the best for YA and the second-best fit for OA.

Overall, both post-hoc hypotheses about *Failed* balancing were supported. The time of logistic transition from a low to high proportion of fall-saving CR commands (${Tmid}_{TLTF}^{CR}$) was temporally closer to a fall (later) in OA than YA; CR commands integrated over the entire range of TLTF (${AUC}_{TLTF}^{CR}$) were less prevalent in OA than YA who fell less; immediately before contacting a fall boundary, OA made a smaller percentage of CR commands (${\%@min}_{TLTF}^{CR}$), and a larger percentage of fall-promoting D commands (${\%@min}_{TLTF}^{D}$) and fall-capitulating I commands (${\%@min}_{TLTF}^{I}$) than YA. Both groups’ command proportions were statistically identical when they had abundant TLTF upon entering the Fall quadrant with identical velocity.

The post-hoc hypothesis of age-dependent, time-critical command switching makes two supplementary predictions: 1) within Fall quadrants, command switching should be better and faster in *Saved* balancing, where falling is averted than in *Failed* balancing, which always ends in a fall, despite identical initial pendulum and command states, and 2) in *Saved* balancing where both groups escape the danger of falling there should be no differences in command switching. The reverse of command percentage as a function of displacement (see Fig. 3) is a rough proxy for percentage as a function of TLTF, and also, it can be calculated for all balancing regimes. Therefore, the regimes can be directly compared in terms of ${AUC}_{P}^{I/CR/A/D}$, ${\%@DOB}_{P}^{I/CR/A/D}$, and ${\theta ext}_{P}^{I/CR/A/D}$. Our analyses, supported both supplementary predictions. For the first question, all three CR variables differed between the *Failed* and *Saved* regimes in fall-saving directions, but *Saved* and *Safe* did not differ. For the second question, all three CR variables were significantly more fall-saving in YA than OA in *Failed* balancing (*p* ≤ 0.004 at least), but there were no such age differences in *Safe* and *Saved* balancing.

**Fig. 5:**
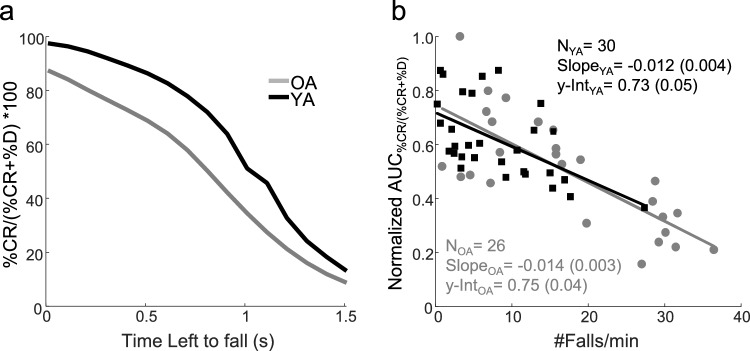
**a** The ratio of fall-saving CR commands to all active commands as a function of TLTF in *Failed* balancing, averaged for OA and YA. **b** Scatterplots of the area under the curve of the plots in panel a (normalized to the highest value in the entire data set) versus the average fall rates per participant, for OA (gray circles) and YA (black squares). Neither the slopes nor y-intercepts of the linear fits differ by age

Figure 5a summarizes the observed coordinated increase in the percentage of fall-saving CR commands and decrease in fall-promoting D commands as TLTF approaches 0. The ratio %CR/(%CR + %D) on the y-axis represents the proportion of fall-saving commands relative to all active commands leading up to a fall. This ratio gets more favorable as falls become more imminent, more dramatically for YA than OA. Including inactivity in the denominator would rescale both curves without altering the age differences because %I as a function of TLTF does not differ by age group, except at the ultimate moment before a fall (${\%@min}_{TLTF}^{I}$), where it would magnify the age differences. This illustrates that **OA are as competent as YA in switching between action and inaction, but OA become more error-prone in choosing which and how much action to take, and they do so later as TLTF approaches zero**. Figure 5b plots the area under composite %CR/(%CR + %D) curves of 5a for each individual on the y-axis as a measure of overall fall-saving behavior against the propensity to fall. The negative slope proves an association between better *Failed* regime command structure (more CR and less D) and better task-level VIP performance (fewer falls). The statistically indistinguishable linear fits for OA (gray circular dots) and YA (black squares) indicate the same relationship holds for both age groups. The distinct cluster of points in the lower right part of Fig. 5b suggests that a subset of “high fallers” may exist—mostly OA but at least one YA—who show extreme dysfunctional coordination of CR and D commands relative to the general population.

*Implications of age-dependent, regime-specific, time-critical switching between balancing command alternatives*. **OA and YA do not differ in task-level performance or command dynamics in *Safe* and *Saved* balancing, they differ only in *Failed* balancing**. In the Safe quadrants, falls are not an immediate threat, and both OA and YA use identical command switching to stay equivalently well around the upright DOB. In the Fall quadrants, the initial pendulum and joystick conditions are the same for both OA and YA until non-optimal CR and D commands lead to an age-dependent divergence of *Saved* and *Failed* balancing. Any mechanistic explanation for the divergence must account for OA and YA being identical in *Safe* balancing, identical in how they escape imminent falls (*Saved* balancing) but different in how they succumb to imminent falls (*Failed* balancing). For example, delayed or less precise encoding of the pendulum state—it’s angular position and velocity—might be evoked to explain the OA deficit in *Failed* balancing but is incompatible with the statistically similar performance of OA and YA in *Safe* and *Saved* regimes. In addition, such regime-specific age differences in switching are improbable if under the sole control of linear optimal feedback mechanisms incorporating delays (Milton et al. 2009a), drift and catch (Milton et al. 2009b). If the parameters of such models were set to reflect age differences in peripheral biomechanical and neurophysiological characteristics, then they should result in age differences in performance independent of the balancing regime instead of the regime-dependent age difference we found. These novel findings complement current notions of human intermittent control, and the key reason for these advances is our novel method of functionally characterizing the phase space rather than treating it as uniform. Furthermore, our demonstrations of age- and regime-specific behavior does not exclude the possibility that models without regime-specific parameterization also operate concurrently.

**Our discovery of the age-dependent logistic structure of I and CR or D command dynamics in *Failed* balancing is consistent with a decision-like process**. A lack of significant difference between OA and YA when there is no imminent threat of falling (*Saved* and *Safe* regimes) implies that balancing may operate in dual decision modes: “oscillate about the DOB” when TLTF is not pressing enough to evoke action, and “fall prevention mode” when under time pressure. In both cases, there could be a logistic, probabilistic decision to act or not, and after the decision to act advances far enough, a choice must be made between CR and D in the Fall quadrants or between CR and A in the Safe quadrants. Such dual, simultaneous modes have been proposed to explain the performance and joystick commands in a MARS self-balancing experiment made difficult by depriving participants of information about self-tilt relative to gravity (Vimal et al. 2019). Zgonnikov and Markkula (2019) previously concluded that decision-making processes that result in transitions from inactivity to corrective action operate when manually controlling a mechanical inverted pendulum. However, they did not model decisions between corrective (CR) versus destabilizing (D) actions in the Fall quadrants or versus anticipatory (A) actions in the Safe quadrants. We note in this context that we have chosen to discriminate recurrent, discrete periods of command “activity/inactivity” without using the term “intermittency,” which often connotes clock-like or phase-dependent switching. In addition, it bears repeating that D commands have not been documented outside the present study and previous MARS studies from our laboratory. It is beyond the scope of this paper to schematize all possible decision transitions, but inspection of Fig. 1 shows that transitions can occur bidirectionally between inaction and action, as well as between the three types of actions (CR, A, and D).

*Relationship of VIP results to bipedal balance and falling, and limitations*. The VIP task is only a partial analog to bipedal balance, and the costs of VIP “falling” are much lower than the cost of real falling. However, the present results provide new insights for investigating falling in at-risk populations. We are unaware of any previous suggestion that falling may be due to frankly destabilizing muscle activation, as opposed to the correct direction being executed too weakly, too late, and/or too intermittently. The VIP paradigm spotlights the continuous, high rate of command switching needed, as well as the high relevance of imminent falling. We are currently examining the possibility that destabilizing commands in the Fall quadrants are confusions with recently activated decision processes in the Safe quadrants, in the VIP task, the MARS self-balancing task, and the real bipedal stance. Such confusions are plausible because they appear in high-rate serial saccade and keyboarding tasks (Rosenbaum et al. 1986; Emeric et al. 2007). Our simple, single-pivot VIP paradigm results have potential parallels to command errors and command switching in bipedal stance. Medial–lateral sway is physically governed by the weight distribution between the two feet and the pressure distribution under each foot (Winter et al. 1996; Rougier 2007), among other factors. Even when one leg gradually assumes the majority of body weight support, it almost instantly engages in contributing a biomechanically disproportionate dominance of the net dynamic center of pressure (while the other leg disengages, acting only as a vertical strut) (Bakshi et al. 2019, 2020). Such concurrent, coordinated command switching is not prominent in the raw center of foot pressure traces but can be decoupled with appropriate two-foot force measurements, and the evolution of different leg muscle command types can be analyzed as a function of the balance regimes defined for the VIP.

In a different vein, the ample literature documenting dual-task, cognitive-postural deficits could be explained by competition between central decision-like balancing mechanisms and dual-task central processing (Muller et al. 2004), but the peripheral postural process would not be expected to compete with secondary cognitive tasks. Finally, it is possible that age differences in command transitions with diminishing TLTF may reflect subjective estimates of risk or fear of falling in the VIP task, which are factors in bipedal fall risk. Our OA participants had less exposure to joysticks than YA participants, but there were no significant differences in fear of falling. In future studies, we are interested in distinguishing the decision-making differences between high fallers, such as those in the lower right of Fig. 5b, and proficient performers, independent of age, in the VIP task, MARS self-balancing, and bipedal stance.

## Significance

This study is the first to identify the higher-order mechanisms of visual inverted pendulum balancing and how aging impacts performance. When the VIP swayed into a state of increased vulnerability to a fall, YA used fall-saving strategies more effectively than OA; OA used the wrong strategy more often and changed to the correct strategy later than YA. Overall, OA balanced like YA but fell differently.

## Data Availability

The data that support the findings of this study are available from the corresponding author upon reasonable request.

## Footnotes

1 Persistent joystick values within ± 1° were included, however quick passage through this band while transitioning the joystick from left to right or vice versa were not included.

2 Normal font denotes a summary variable (area under the curve in this case) of the profile of command type percentage across a balancing regime; the subscript denotes whether the summary statistic is computed on the profile of either command type with respect to pendulum Position (P) or TLTF (in this case position); the superscript abbreviates which command type dynamics is summarized.

3 The results are similar when the maneuver percentages are plotted as a function of pendulum velocity.

4 Note that the pendulum can be in proximity to boundary in all three regimes, if the velocity becomes inward towards the DOB then it enters the *Safe* regime, while in the *Saved* and *Failed* regimes it is outward, towards the boundary.
